# Morphology and Hemodynamics of Cerebral Arteries and Aneurysms in a Rare Pair of Monozygotic Twins

**DOI:** 10.3390/diagnostics13122004

**Published:** 2023-06-08

**Authors:** Hang Yi, Zifeng Yang, Luke C. Bramlage, Bryan R. Ludwig

**Affiliations:** 1 Department of Mechanical and Materials Engineering, Wright State University, Dayton, OH 45435, USA; 2 Division of NeuroInterventional Surgery, Department of Neurology, Wright State University/Premier Health—Clinical Neuroscience Institute, 30E Apple St., Dayton, OH 45409, USA; lcbramlage@premierhealth.com (L.C.B.); brludwig@premierhealth.com (B.R.L.); 3 Boonshoft School of Medicine, Wright State University, Dayton, OH 45435, USA

**Keywords:** identical twins, configuration, blood flow characteristics, environmental and genetic factors, neurovascular diseases

## Abstract

In this preliminary study, the underlying pathophysiology mechanisms of cerebral aneurysms (CAs) in monozygotic twins (MTs) were investigated via a rare pair of MTs (twin A and twin B) involving four reconstructed arterial models using preclinical information. First, dimensions and configurated outlines of three-perspective geometries were compared. Adopting an in-vitro validated numerical CA model, hemodynamic characteristics were investigated in the MTs, respectively. Despite expected genetic similarities, morphological comparisons show that configurations of cerebral arteries exhibit significant differences between the twins. The ICA size of twin A is larger than that in twin B (2.23~25.86%), varying with specific locations, attributing to variations during embryological developments and environmental influences. Numerical modeling indicates the MTs have some hemodynamic similarities such as pressure distributions (~13,400 Pa) and their oscillatory shear index (OSI) (0~0.49), but present significant differences in local regions. Specifically, the difference in blood flow rate in the MTs is from 16% to 221%, varying with specifically compared arteries. The maximum time-averaged wall shear stress (53.6 Pa vs. 37.8 Pa) and different local OSI distributions were also observed between the MTs. The findings revealed that morphological variations in MTs could be generated by embryological and environmental factors, further influencing hemodynamic characteristics on CA pathophysiology.

## 1. Introduction

According to the fact sheets from the World Health Organization (WHO), blood and vascular associated diseases (e.g., heart diseases, stroke, hypertensive disease, aneurysm) have become the top global factor of death; nearly 5% of the population in the United States have at least one cerebral aneurysm (CA) and about 0.2% result in stroke from ruptures, annually [1,2,3]. Both genetic and environmental factors can contribute to the initiation/growth of intracranial aneurysms; however, the impact of each factor is still ambiguous [3,4]. Uniquely, hemodynamic roles in monozygotic twins (MTs) provide a scenario limiting the variability of genetic factors. Thus, it is desirable to reveal the underlying mechanisms associated with morphological alterations and hemodynamic differences affected by embryological and external environmental factors utilizing MTs’ neurovascular systems for investigations.

Up to date, there is an incomplete understanding of the importance of many measurable stresses in the cerebral vascular anatomy associated with MTs [5], although numerous studies have demonstrated the potentiality of low wall shear stress (WSS) leading to different tissue mechanical stabilities of the arterial wall, as well as regions of accelerating blood flow with high WSS and positive WSS spatial gradients leading to extracellular matrix degradation and cellular loss, involving the investigations on various factors such as blood flow shunting ratios in bifurcated arteries, morphological variations of arteries, growth and rupture of small CAs, post-treatments of CAs [6,7,8,9], etc. Others have proposed arterial regions subjected to high WSS, but negative WSS spatial gradients, appear to be protected from matrix degradation [10]. The expression of specific endothelial cell and smooth muscle cell phenotypes appears to depend not only on an interplay of local hemodynamic environmental factors, but also on intrinsic genetically programmed biology [11,12]. MTs own the unique features that reduce genetic variability (e.g., deoxyribonucleic acid (DNA)) and often share similar chronic health problems and habits, which is due to the fact that the identical twins grow from the fertilization of a single egg by a single sperm, with the fertilized egg then dividing into two. Arterial morphologies and associated blood flow patterns can be affected by post-zygotic changes and epigenetic effects in utero which are not completely understood and still need to be identified comprehensively. The assumption of identical morphology and hemodynamics for MTs is questionable, and how this assumption can lead to hemodynamics differences in the associated CAs needs further investigation quantitatively.

However, to the best of our knowledge, there is no existing study to investigate the performances of hemodynamics such as the WSS and oscillatory shear index (OSI) concerning CAs in the MTs. The specific study [13] compared the blood flow rates in the circle of Willis (CoW) and CoW morphology in sixty-four twins (i.e., 19 MTs and 13 dizygotic twins) using transcranial color-coded sonography, which is similar to a different study [14] which included more MT cases. The difference of CoW morphologies between twins was observed while the specific hemodynamic features (e.g., OSI and WSS) were not investigated. Another study [15] involved detailed studies in configurations and hemodynamics of CoWs of six pairs of MTs while not associated with CAs. Other studies focused more on reporting CA discoveries in MTs based on scanned digital subtraction angiography (DSA) images [16,17,18,19,20,21,22,23,24] rather than investigating specific hemodynamic factors resulting in CAs in the corresponding arteries and CAs. Thus, to facilitate the understanding of morphology and hemodynamics in CAs involved in the MTs, and to enrich the case studies in the CA research community, a careful study on the pathophysiology of CAs in MT models is extremely valuable.

**Figure 1:**
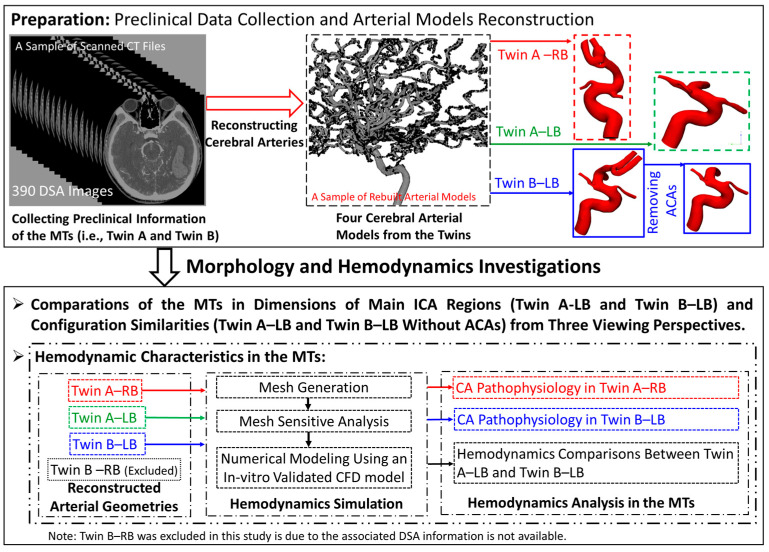
The flowchart of this work to investigate the morphology and hemodynamics of cerebral arteries and aneurysms in the MTs.

To partially address the abovementioned concerns, a comprehensive case study in this paper was conducted to quantify the differences in morphology and hemodynamics of cerebral arterial and aneurysmal models in MTs (i.e., twin A and twin B), specifically. As shown in Figure 1, four cerebral arterial and aneurysmal models in a rare pair of MTs (i.e., twin A-left brain (LB), twin B-right brain (RB), twin B-LB, and twin B-LB without anterior cerebral artery (ACA) branches) were reconstructed using the preclinical DSA images of the MT patients. Then, the configurations and dimensions of the reconstructed models were compared to identify differences related to embryological and environmental factors in a rare pair of MTs. To quantify hemodynamic characteristics in the MTs, an in-vitro validated computational fluid dynamics (CFD) CA model [25] was employed to model hemodynamic characteristics, such as time-averaged WSS (TAWSS), OSI, and time-averaged pressure (TAP), in the MTs. The findings provide a pathway to understand the impacts of genetic and environmental factors on morphology which can further affect hemodynamics-affiliated pathogenesis of neurovascular diseases. In addition, this study reveals the irrationality of the hypothesis that MTs share the same morphologies of cerebral arteries and aneurysms, since it could lead to significant errors in evaluating the hemodynamic risks to the pathophysiology of CAs in the MTs under such an assumption.

## 2. Materials and Methods

### 2.1. Information and Symptoms of Patients

**Figure 2:**
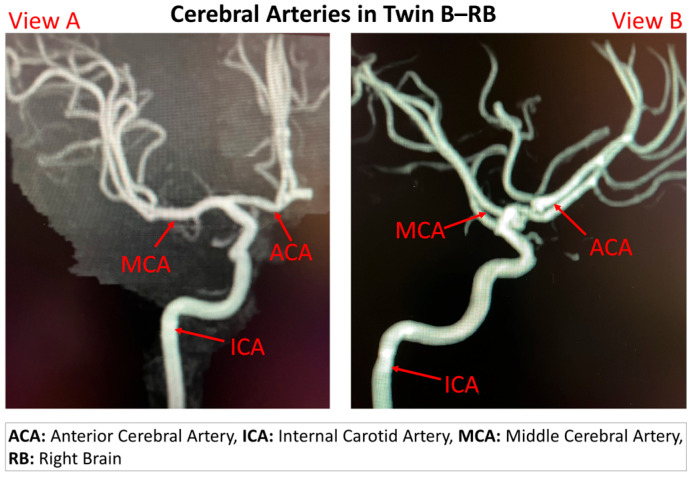
Cerebral artery system in twin B-RB based on collected MRI images from two viewing perspectives (i.e., view A and view B).

The pair of male MTs is 45~55 years old, with an identical appearance and height, apart from the weights (twin A with 87.3 kg and twin B with 70.3 kg). Both twins had similar comorbidities (nearly identical smoking histories and essential hypertension profiles) according to the acquired information from a local hospital (Dayton, OH, USA). Twin A suffered a ruptured CA located at the bifurcated region of ACAs in the right brain, while he has no left ACA branches. Then, physicians suggested the twin brother (twin B) of twin A to do a screening for CAs. Twin B has a small and unruptured bleb located at the left ACA bifurcation. However, it should be mentioned that digital subtracted angiographic information for twin B-RB is not available because no aneurysm was found in the right brain, but magnetic resonance imaging (MRI) of cerebral arteries showed that twin B-RB has ACAs, as shown in Figure 2.

### 2.2. Reconstructed Arterial and Aneurysmal Models

**Figure 3:**
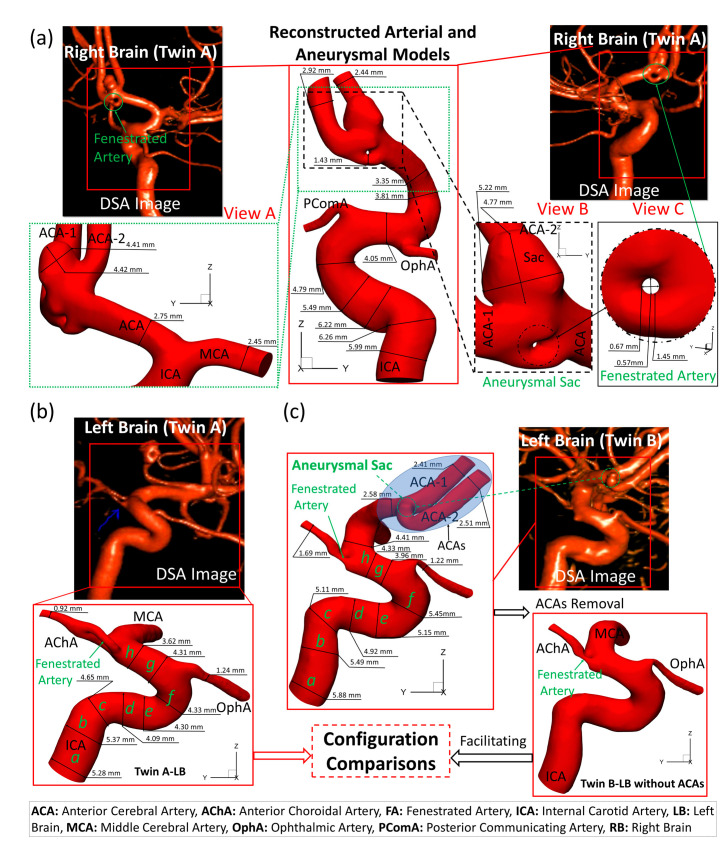
Morphologies and dimensions of cerebral arteries and aneurysms in the MTs. (**a**) Specific arterial configurations and dimensions in twin A-RB, with an aneurysmal sac (~5 mm) (view B) and a fenestrated artery (view C); (**b**) Dimensions and configuration of the arteries in twin A-RB; (**c**) Complete arterial and aneurysmal geometries with a small aneurysm bleed located at the ACA bifurcation (left of (**c**)) in twin B-LB, and an arterial model (i.e., right of (**c**)) with ACAs virtually removed based on the left complete model to facilitate the morphological comparisons in the MTs.

As shown in Figure 3, the reconstructed four models based on digital subtraction angiography (DSA) images, i.e., twin A-RB, twin A-LB, twin B-LB, and twin B-LB with ACA branches virtually removed (only for the purpose of configuration comparisons), were reconstructed using Mimics Research 23.0 (Materialise NV, Leuven, Belgium), based on 390 non-invasive scanned DSA images for each brain by the Artis Zee system (Siemens Medical Solutions USA, Inc., Malvern, PA, USA). To cover the volume of cerebral arteries, the scanning was conducted with a collimated section width of 1 mm, a pitch of 1.5, and a gantry rotation time of 0.5 s in a total of 9 s. To balance arterial curvature and smoothness, reducing tolerance 0.03 with 5 iterations and smooth factor 0.4 with 10 iterations were adopted in the reconstruction. Reconstructed models were used for comparisons of morphologies and hemodynamics between the twins.

### 2.3. Numerical Modeling

#### 2.3.1. Geometry and Mesh

**Figure 4:**
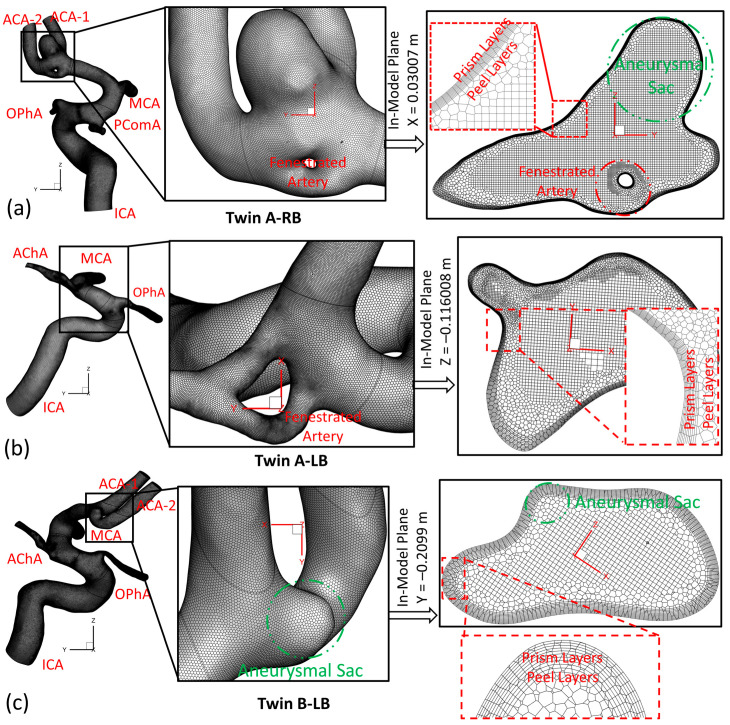
Detailed poly-hexcore mesh details of the MTs for numerical simulations. (**a**) Twin A-RB; (**b**) Twin A-LB; (**c**) Twin B-LB.

To investigate hemodynamic characteristics in the rare pair of MTs, e.g., twin A and twin B in this study, three arterial and aneurysmal models, e.g., twin A -LB and -RB, and twin B-RB, were reconstructed using scanned preclinical images provided by a local hospital (Dayton, OH, USA), as mentioned in Section 2.1. Using ANSYS fluent meshing 2022 R2 (ANSYS Inc., Canonsburg, PA, USA), a total of nine polyhexcore meshes (i.e., mesh 01–09) with different meshing sizes (see Table 1) were created for mesh independence tests, and meshing details have been visualized in Figure 4a–c for twin A-RB, twin A-LB, and twin B-LB, respectively. To decide the final/prime mesh for hemodynamics modeling, the mesh independence test has been performed under steady-state blood flow by considering the optimized balance between computational accuracy and efficiency via ANSYS fluent 2022 R2 (ANSYS Inc., Canonsburg, PA, USA). 

**Figure 5:**
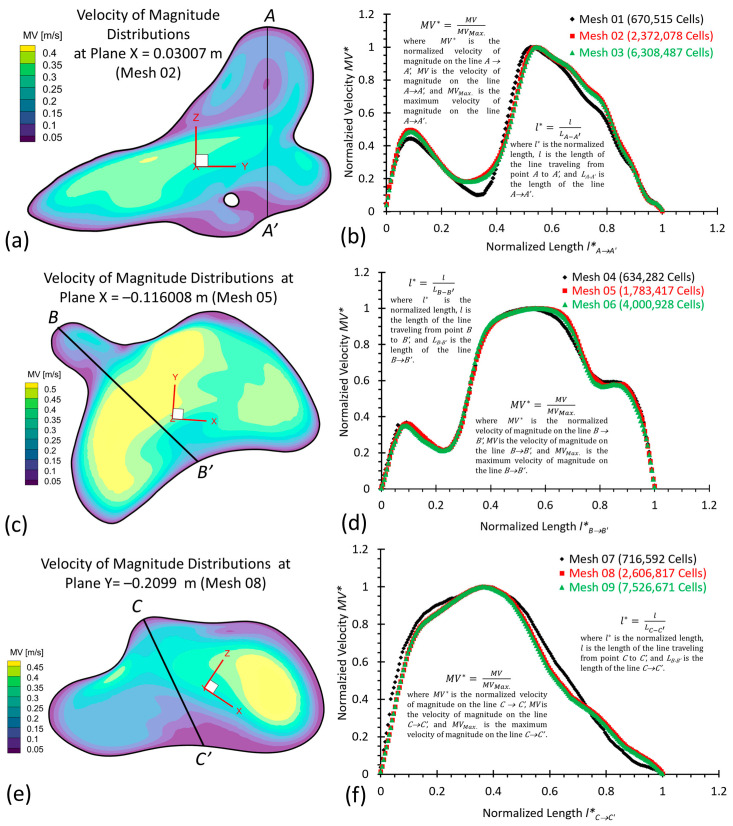
Mesh independence tests for the models of MTs. (**a**) Velocity distribution contours at plane X = 0.03007 m in mesh 02; (**b**) Mesh independence test by comparing non-dimensionalized velocity profiles at line *A*–*A′* among mesh 01, mesh 02 (final), and mesh 03; (**c**) Velocity distribution contours at plane X = −0.116008 m in mesh 05; (**d**) Mesh independence test by comparing non-dimensionalized velocity profiles at line *B*–*B′* among the mesh 04, mesh 05 (final), and mesh 06; (**e**) Velocity distribution contours at plane Y = −0.2099 m in mesh 08; (**f**) Mesh independence test by comparing the non-dimensionalized velocity profiles at line *C*–*C′* among the mesh 07, mesh 08 (final), and mesh 09.

Specifically, regions (i.e., aneurysmal sac/bleb and arterial walls) with critical hemodynamic characteristics were designated as refined mesh elements. Mesh independence studies were conducted by comparing the non-dimensionalized velocities $V_{i–i^{\prime }}^{*}$ at designated lines across the models (i.e., Figure 5a–f), i.e., *A*–*A′*, *B*–*B′*, and *C*–*C′* in the three investigated cerebral arterial models with in-model planes (i.e., plane X = 0.03007 m in twin A-RB, plane X = −0.116008 m in twin A-LB, and plane Y = −0.2099 m in twin B-LB), respectively. A mean blood flow rate of 265 mL/min [26] in the internal carotid artery (ICA) was employed to investigate the mesh sensitivity analysis in the three rebuilt models. In addition, a blood density of 1050 kg/m^3^ and blood viscosity of $3.5\times 10^{-3}$ kg/m·s were adopted for the mesh sensitive analysis in this study.

Equations for the normalized velocity $MV^{*}$ and normalized length, i.e., $\;L_{i–i^{\prime }}^{*}$ and $L_{i^{\prime \prime }–i^{\prime \prime }}^{*}$, are defined by:

(1) $$MV^{*}=\frac{MV}{MV_{Max.}}\;$$

(2) $$l_{i–i^{\prime }}^{*}=\frac{l}{L_{i–i^{\prime }}}\;$$

where $MV_{Max.}$ is the maximum magnitude of velocity along selected lines *A–A′*, *B–B′*, and *C–C′* shown in Figure 5a,c,e, respectively. MV is the magnitude of velocity along selected lines from $A\to A^{\prime }$, $B\to B^{\prime }$, and $C\to C^{\prime }$ of cut-planes (i.e., plane X = 0.03007 m, plane X = −0.116008 m, and plane Y = −0.2099 m), respectively. l denotes the length of the location of velocity vectors traveling from *A* to *A′*, *B* to *B′*, and *C* to *C′*, accordingly. $L_{i–i^{\prime }}$ are the lengths of lines *A–A′*, *B–B′*, and *C–C′*, respectively.

Using different generated meshes (see Table 1), the comparisons of non-dimensionalized velocity profiles are shown in Figure 5b,d,e for the corresponding models, e.g., twin A-RB, twin A-LB, twin B-LB, respectively. Mesh 01, 04, and 07 for the corresponding model are too coarse to secure precise results. The differences in the calculated velocities are smaller than 0.5% between mesh 02 and mesh 03. The same results can also be obtained when comparing mesh 05 and mesh 06, and mesh 08 and mesh 09, respectively. Therefore, with 10 prism layers, 3 peel layers, and a sizing growth ratio of 1.05 to capture accurate hemodynamic features around arterial walls, the three final meshes (i.e., mesh 02, mesh 05, and mesh 08) have 2,372,078, 1,783,417, and 2,606,817 cells for the models of twin A-RB, twin A-LB, and twin B-LB to investigate the accurate hemodynamic characteristics, respectively.

#### 2.3.2. Mathematical Equations

**Figure 6:**
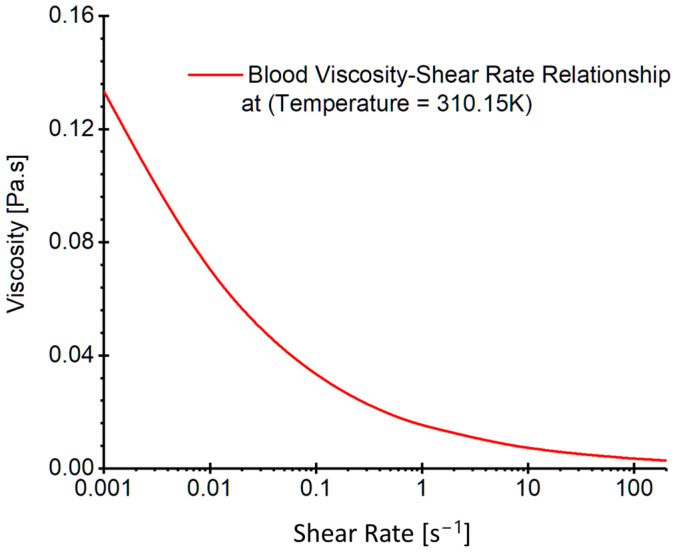
Realistic non-Newtonian blood viscosity profiles employed in the CFD modeling [25].

To explore hemodynamic patterns in MT models, our in-vitro validated non-Newtonian CFD CA model [25] was employed to simulate the blood flow regime. The unsteady and periodic pulsatile, incompressible blood flow can be modeled by the continuity and momentum equations, which are, i.e.,

(3) $$\nabla \cdot \vec{v}=0$$

(4) $$\frac{\partial \vec{v}}{\partial t}+\rho \nabla \cdot \left(\vec{v}\vec{v}\right)=-\nabla p+\nabla \cdot \left(\mu \left(\nabla \vec{v}+{\left(\nabla \vec{v}\right)}^{T}\right)\right)+\rho \vec{g}$$

where $\vec{v}$, p, $\vec{g}$, and ρ are the velocity vector, pressure, gravity vector, and blood density, respectively. In this study, a blood density of 1050 kg/m^3^ is employed for hemodynamic modeling, and blood viscosity profiles (see Figure 6) were determined by a non-Newtonian viscosity model at a normal body temperature of 310.15 K. Such a profile was plotted based on the blood viscosity model which considered blood non-isothermal features and shear-thinning properties in our previous publication [25]. Specifically, the model is expressed by, i.e.,

μ={(5a)μmin γ˙≥γ˙max(5b)aγ˙b−1  (γ˙min<γ˙<γ˙max)(5c)μmax  γ˙≤γ˙min

In Equation (5a–c), a is the consistency index registered as 1.51 $\times 10^{-2}\;\mathrm{Pa}\cdot s^{b}$, and b is the power-law index defined as 0.685. Additionally, ${\dot{\gamma }}_{min}$ is the minimum shear rate, and ${\dot{\gamma }}_{max}$ is the maximum shear rate. In this study, in accordance with our previous publications [25], $3.0\times 10^{-3}\;\mathrm{Pa}$·s and $1.5\times 10^{-1}\;\mathrm{Pa}\cdot s$ were designated for $\mu_{min}$ and $\mu_{max}$, respectively. In Equation (5), shear rate $\dot{\gamma }$ in the modeling can be calculated by:

(6) $$\dot{\gamma }={\left(\frac{1}{2}\nabla \vec{v}\;:\nabla \vec{v}\right)}^{\frac{1}{2}}$$

Within the neurovascular system, the pulsatile blood flow domain presents laminar-to-turbulence features. Thus, this study employed the shear stress transport (SST) k−ω turbulence model [27] to predict the “laminar-to-turbulent” transition flowing. Additionally, the low Reynolds correction was adopted to hemodynamic characteristics more accurately in the near-wall domains with a possible low Reynolds number. Such a correction is a result of the combination of the employed turbulence model, which was coupled with two parameters, i.e., the correlation coefficient $\alpha^{*}$ and turbulent Reynolds number $Re_{t}$ [28]. Specifically, the equations are, e.g.,

(7) $$\mu_{t}=\alpha^{*}\frac{\rho k}{\omega }\;$$

(8) $$\alpha^{*}=\frac{0.025+\frac{{Re}_{t}}{6}}{1+\frac{{Re}_{t}}{6}}\;$$

(9) $$Re_{t}=\frac{\rho k}{\mu \omega }\;$$

In Equations (7)–(9), $\mu_{t}$, k, and ω are the turbulent viscosity, turbulence kinetic energy, and specific dissipation rate, respectively. In addition, the hemodynamic parameters, such as TAWSS, OSI, and TAP, were introduced and employed in our previous study [8].

#### 2.3.3. Initial and Boundary Conditions 

**Figure 7:**
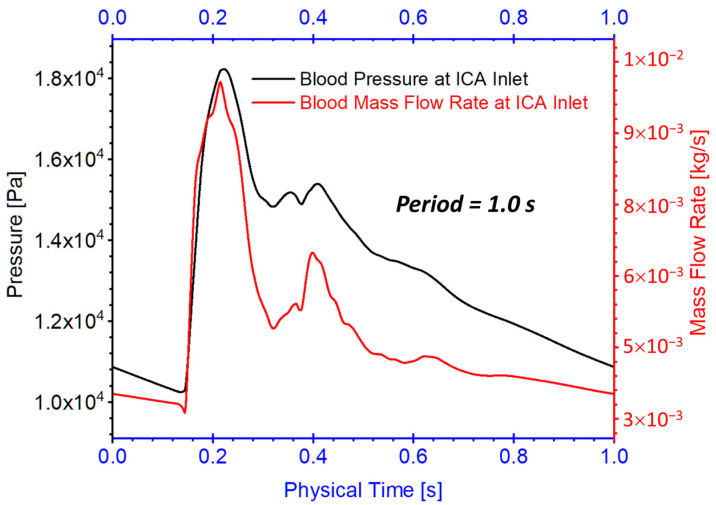
Blood flow rate and pressure waveforms for ICA inlet boundary condition in the twins (i.e., models of twin A-RB, twin A-LB, and twin B-LB).

Three arterial models in twin A-RB, twin A-LB, and twin B-LB were employed to investigate hemodynamic characteristics in the MTs. Specifically, one pulsatile flow rate waveform with a period of 1.0 s obtained from the 1D model [7,8,29] was adopted as the flow rate conditions at the ICA inlet (see Figure 7), mimicking pulsatile blood flow conditions. The pressure outlet was assumed at outlets flow condition, i.e., ophthalmic artery (OphA), posterior communicating artery (PComA), anterior choroidal artery (AChA), ACA, resighted with a gauge pressure of 0. The backflow direction at the outlets was determined by the flows in the adjacent cells. Additionally, the stationary, non-slip, and no-penetration condition was assumed for the arterial walls.

#### 2.3.4. Numerical Setups

The numerical modeling work was performed by Ansys fluent 2022 R2 (Ansys Inc., Canonsburg, PA, USA) on an HP^®^ Z840 workstation onsite, with specifications of two Intel^®^ Xeon^®^ E5-2687W-V4 Processors and 128 GB RAM. Under the designated time step-size of $1\times 10^{-4}$ s, it cost ~71, ~54, and ~ 75 h to finish the modeling with three physical periods (i.e., 3 s) for the models of twin A-RB, twin A-LB, and twin B-LB, respectively. The simulations of three pulsatile cycles were to ensure the modeling stability, and the results under the third pulsatile period (i.e., period = 1.0 s) were used to investigate hemodynamic characteristics in the MT models. In addition, pressure-implicit with splitting-of-operators algorithms were adopted for the pressure-velocity coupling, and least-squares cell-based schemes were employed to compute cell gradients. The second order scheme was employed for discretizing pressure. The discretization of momentum, turbulent kinetic energy, and the specific dissipation rate was designated to use the second-order upwind scheme. Once the residual was less than $1.0\times 10^{-4}$, the calculations were converged for continuity, momentum, and additional equations.

## 3. Results

### 3.1. Comparisons in Dimensions and Configurations

Figure 3a,b manifests that the ICA diameter of twin A is 10% larger than the counterpart in the LB, and RB has a fenestrated artery (FA) with an inner diameter of about 0.6 mm with an aneurysmal sac (~5 mm) at the ACA bifurcation. Both twin A and twin B have the same AChA branches with a fenestration near its origin, but neither of them has a PComA, as shown in Figure 3a,c. Unlike the configuration in twin A-RB, twin B-LB does not have a FA complex at the ACA bifurcation; however, there is a small unruptured CA bleb (~1 mm) associated with the left ACA bifurcated complex. More interestingly, twin A-LB does not have a left ACA which is completely different from twin B-LB. More specifically, the dimensional comparisons in the main ICA were analyzed with eight designated locations, i.e., lines *a*, *b*, *c*, *d*, *e*, *f*, *g*, and *h*, in a perspective view on plane YZ (see Figure 3b,c), and the quantitative variations were calculated as shown in Table 2. Specifically, the relative difference in the diameter between twin A and twin B was calculated using Equation (10), i.e.,

(10) $$Relative\;Difference=\frac{D_{Twin\;B}-D_{Twin\;A}}{D_{Twin\;A}}\times 100\%\;$$

In Equation (10), $D_{Twin\;A}$ and $D_{Twin\;B}$ are the ICA diameters of the MTs in the compared locations (see Figure 3b,c), respectively. Following the blood flow direction, Table 2 shows that except for one compared location, the size of the main ICA regions in twin B is larger than the size in twin A, varying from 2.23% to 25.86%, specifically. The exclusive comparison in ICA, i.e., line g (see Figure 3b,c), is close to the connected region with the OphA, registering the relative difference of −8.12%.

**Figure 8:**
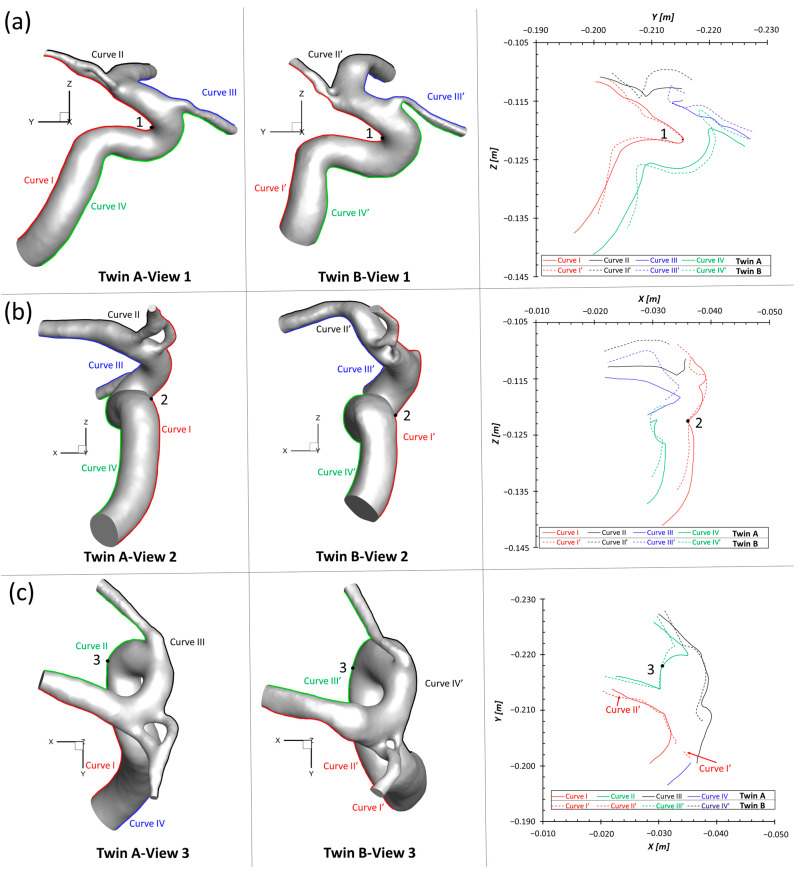
Comparison of projective graphs and outline curves of partial LB arteries in the MTs in the Cartesian coordinate system. (**a**) Left two projective graphs show the projective images on the YZ-plane, and corresponding outline curves (right) show the lineaments of LB arteries in twin A and twin B with the overlapped benchmark point 1 (apex of the curvature); (**b**) Left two projective graphs show the projective images on the XZ-plane, and corresponding outline curves (right) show the lineaments of LB arteries in twin A and twin B with the overlapped benchmark point 2; (**c**) Left two projective graphs show the projective images on the XY-plane, and corresponding outline curves (right) show the lineaments of LB arteries in twin A and twin B with the overlapped benchmark point 3.

To match the extracted portion (ICA mainly) of the arterial model in twin A-LB and avoid blocking the view of MCA branches, the ACAs in twin B were removed (right corner of Figure 3c) purely for geometric comparisons. Specifically, three sets of projective images (i.e., view 1, view 2, and view 3) and associated outlines (i.e., curve I and curve I′, curve II and curve II′, curve III and curve III′, and curve IV and curve IV′) (see Figure 8) of the geometries from three perspective directions (YZ-plane, XZ-plane, and XY-plane) were selected for comparisons, with the corresponding benchmarked point, 1, 2, and 3, respectively. It can be found that although twin A and twin B show similar outlines at local regions near the benchmarked point, most parts of the cerebral arterial configurations show subtle differences from each other significantly.

### 3.2. Hemodynamics in Twin A-RB

**Figure 9:**
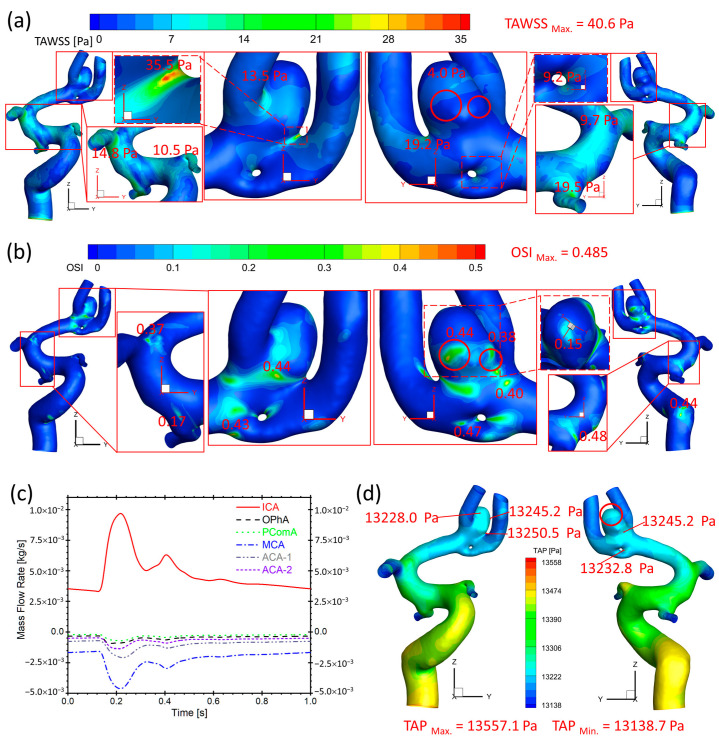
Hemodynamic characteristics in the arterial and aneurysmal models in twin A-RB. (**a**) Specific TAWSS distributions on the model walls in which relatively high TAWSS locations were enlarged and highlighted; (**b**) Detailed OSI distribution contours on the model walls in which relatively high OSI locations were enlarged and highlighted; (**c**) Mass flow rates at ICA, OphA, PComA, MCA, ACA-1, and ACA-2 along the blood cardiac flow period (1.0 s); (**d**) TAP distributions on the arterial walls in which the maximum and minimum TAP, and some local sites with relatively high TAP, were marked.

Figure 9a presents that the ACA bifurcation region in twin A-RB registers relatively larger TAWSS than other surrounding arteries. Especially, the aneurysmal neck regions register higher TAWSS than other regions, reaching up to 35.5 Pa. Figure 9b manifests relatively high OSI distributions, which also appear on bifurcated regions and aneurysmal neck regions (close to 0.45) where flow diverts as the blood stream approaches such locations. In Figure 9a,b, the low TAWSS (less than 4.0 Pa) and high OSI (higher to 0.44) circumstances can be discovered in highlighted regions with red circles, which could be an essential factor on the growth and rupture of the aneurysmal sac in the ACA bifurcation of twin A-RB.

As for the blood flow rate in each artery (see Figure 9c), it has a close relationship with the sizes of the corresponding arteries which are close to the shunting location from the main ICA. The MCA has the largest diameter among bifurcated arteries which dominates ~50% of blood shunts from the ICA, while PComA is the smallest artery which shunts the least blood flowrate (~5% of total flowrate). There is no significant difference in TAP distributions (~13,250 Pa) (see Figure 9d) on the artery walls near ACAs compared to the averaged pressure (13,158 Pa) of the ICA inlet (black waveform in Figure 9b).

### 3.3. Hemodynamic Comparisons: Twin A-LB vs. Twin B-LB

**Figure 10:**
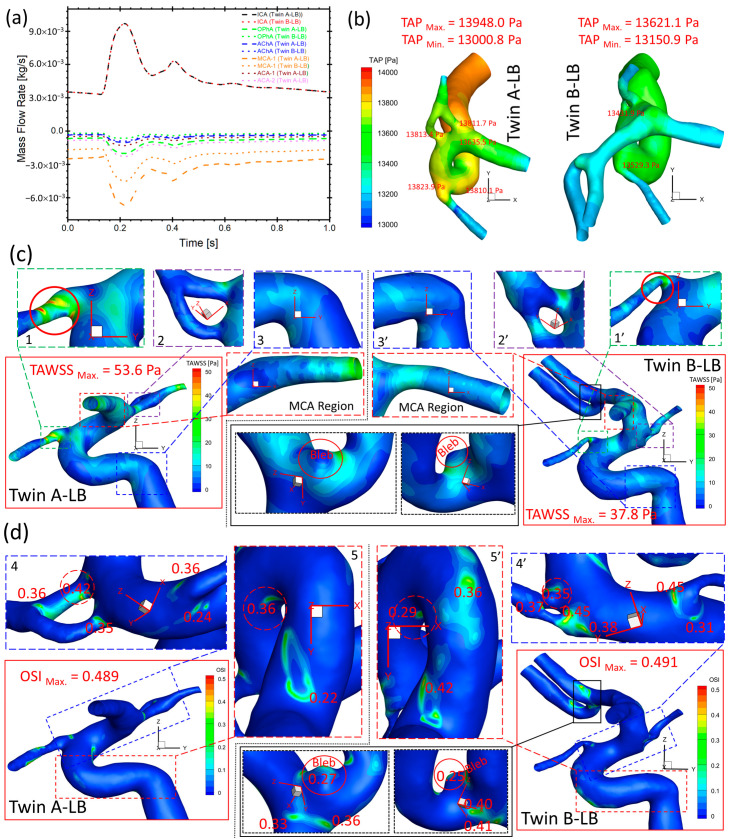
Comparisons of hemodynamic characteristics in the arterial and aneurysmal model between twin A-LB and twin B-LB. (**a**) Mass flow rates at ICA, OphA, AChA, MCA, ACA-1, and ACA-2 along the blood cardiac flow period (1.0 s); (**b**) TAP distributions on arterial walls in one cardiac pulsatile period (1.0 s); (**c**) Specific TAWSS distributions on the arterial and aneurysmal bleb walls; (**d**) Detailed OSI distribution contours on the arterial and aneurysmal bleb walls.

Figure 10a presents that the bifurcated arteries with a larger diameter shunt more flows from the ICA in both twin A-LB and twin B-LB arteries, similar to the case in twin A-RB (see Figure 9c). However, significant differences in shunts in corresponding arteries (i.e., OphA, AChA, and MCA) were observed in the twins, with discrepancies of the volumetric blood flow rate in corresponding arteries between the MTs, from 16% (smallest) in AChA to 221% (largest) in the OphA, respectively. The blood flowrate in bifurcated twin A-MCA (without left ACAs) is much higher than the counterparts in twin B, because twin B-ACAs shunt over 30% of the flowrate from the ICA. 

Under the identical flow boundary condition at the ICA inlet of the MTs, TAWSS on most regions of the twin A arterial wall is higher than the shear stress in twin B (Figure 10c), similar to the observations of higher TAP distributions in twin A (Figure 10b), registering 13,000~13,948 Pa in twin A-LB and 13,151~13,621 Pa in twin B-LB, respectively. The twins registered the highest TAWSS on the same artery (i.e., OphA) with significant differences in specific locations and absolute values, e.g., 53.6 and 37.8 Pa (regions 1 and 1′ in Figure 10c), respectively. The small difference of TAWSS distributions can also be found in 2 and 2′ (FA regions of the AChA domain), and 3 and 3′ (ICA region) in Figure 10c, separately. These regions register relatively low TAWSS with less than 10 Pa, which also can be discovered in aneurysmal bleb regions in twin B-LB. Moreover, MCA regions present completely different characteristics in TAWSS distributions between twin A and twin B (see Figure 10c), in which twin B shows higher TAWSS in the entrance region than the distal end, while twin A shows the reversed distribution pattern. In contrast to TAWSS comparisons, OSI values in twin A are generally smaller than counterparts in twin B on most regions except two highlighted regions (regions 4 and 4′ in the AChA, and 5 and 5′ in the ICA), shown in Figure 10d, while the registered highest OSI in twin A (0.489) and twin B (0.491) are similar. Similar to the TAWSS comparisons, the twins present locations with a relatively high OSI (regions 4 and 4′, and 5 and 5′ in Figure 10d) on the same artery, while specific locations and associated marked values are different. Additionally, a small bleb in the bifurcated left ACAs of twin B-LB (highlighted in Figure 10c,d) suffers a low TAWSS and relatively high OSI under the pulsatile cardiac flow conditions. OSI values on the bleb dome are small (less than 0.1), while neck regions are relatively high, reaching 0.27.

## 4. Discussion

### 4.1. Similarities of Morpholgies and Hemodyanmics

The results in Section 3.1 and Section 3.3 show that there exist significant differences between the investigated MTs in terms of morphologies and hemodynamic characteristics. The dimensions and morphology of the neurovascular systems are not identical because of gene mutation, post-zygotic changes in DNA, epigenetic effects, or other external environmental factors, which might influence overall gene expression. As shown in Figure 3, twin A-LB has no ACAs, which is similar to the findings in a previous report [13] that in terms of morphologies. The fact is that one of the twins is most likely missing ACAs, which may affect the ICA size as we found the diameters of ICA differ from each twin distinctly (see Figure 3b,c)). Such influences of missing ACAs in twin A on the ICA sizes need more case/statistical studies for further comparisons.

It is worth mentioning that hemodynamic patterns can be affected dominantly by configurations and dimensions of cerebral arteries. For instance, twin A-LB and twin B-LB present different TAWSS performances on marked MCA regions shown in Figure 10c, which is because the MCA diameter shrinks from the junction end (entrance) to the distal end in twin A, but slightly expands in twin B. This difference then influences the velocity gradient in the artery and finally results in different TAWSS distributions. Additionally, the different sizes in the cerebral arteries lead to variations in TAP loss. The TAP loss in twin A is higher than the corresponding TAP loss in twin B (see Figure 10b), although differences in absolute values are insignificant, which is due to the fact that the dynamic pressure is insignificant to affect the total blood pressure intrinsically [8,25]. However, it is worth mentioning that the real flow conditions (flow rate and pressure) may be different from the current setup (assumed identical) since the ICA flow conditions could not be identical in the twins. Additionally, the gauge pressure zero strategy may cause different blood rates with the real physiological conditions. While the patient-specific flow conditions were not available in the current study, the future plan is to employ the Doppler ultrasound technique [30] to measure the patient-specific blood flowrate in the ICA and associated outlets in follow-up examinations, to facilitate more accurate hemodynamic information through CFD modeling.

Therefore, although the investigated MTs share the same DNA inherited from their parents, the gene mutation and/or embryological factors (post-zygotic changes in the DNA and epigenetic effects that might influence overall gene expression) could significantly affect the morphological sizes and configurations in the cardiovascular and neurovascular system, such as no ACAs in twin A-LB. As a result, the variations in morphologies and blood flows in the neurovascular system can lead to varying hemodynamic performances on the arterial walls, then further manifesting various CA symptoms.

### 4.2. Hemodynamic Effects on CA Pathophysilogy

The partial aneurysm neck regions in twin A-RB suffer larger TAWSS than other local regions (see Figure 9a) due to the direct impingements by blood flow, which have been reported in previous investigations [8,25]. These results indicate that the local neck region of the current CA in twin B-RB may have potential risks to form small or secondary aneurysms/blebs, which is in line with the hypothesis that the large WSS integrated with a positive TAWSS gradient could trigger a mural-cell-mediated manner that could be associated with the generation, progression, and rupture of small or secondary aneurysm phenotypes [8,31,32]. However, in fact, the aneurysmal sac in twin A was ruptured, possibly due to distinct hemodynamic characteristics that low TAWSS and high OSI circumstances were registered on the dome during the cardiac flow conditions, highlighted with red circles (see Figure 9a). Such luminal surfaces could become the enlargement and then rupture locations in the CA by causing inflammatory-cell-mediated destructive reshaping [32]. In terms of the aneurysmal bleb in twin B-LB, it has the potential to rupture since this bleb suffers a low TAWSS and relatively high OSI (see Figure 10d) repeatedly during blood circulation as well as the strong flow impingement; thus, surveillance should be proceeded due to its small size but with considerable probability to enlarge and rupture.

In addition, a FA complex near the ACA bifurcations of twin-RB has been found. The specific influences of such a structure contribute to the generation, growth, and rupture of the aneurysmal sac was investigated in another research work which is under peer-reviewing.

### 4.3. Limitations and Future Plans

It needs to be mentioned that this study solely focused on comparisons in morphologies and hemodynamics in cerebral arteries of a rare pair of MTs which are associated with the pathophysiology of CAs directly, while more cases will be involved to conduct a detailed statistical study in following-up examinations of our future plan, via the long-term academic relationship with Miami Valley Hospital of Premier Health in Dayton, Ohio. Additionally, as mentioned in Section 4.1, the flow conditions for the inlet and outlets may have differences from the real physiological conditions in the MTs, and the settings of the zero-gauge pressure at the outlets may not be a prime strategy for outlet boundary conditions. However, real blood flow conditions in the cerebral arteries were not available in the current study; our future plan is to use the Doppler ultrasound technique [30] to measure the patient-specific blood flowrate in concerned arteries in follow-up examinations, to facilitate more accurate hemodynamic simulations. DSA information for twin B-RB is not available currently, which limited comparisons between twin A-RB and twin B-RB. Once we obtain the DSA information for twin B-RB, a more complete study with physiological boundary conditions will be conducted in the future. Additionally, this study did not incorporate arterial compliance into the simulation (e.g., the deformation effects between the arterial wall and the blood stream); although many studies involved fluid–structure interaction (FSI) between the arterial wall and blood for CA studies [33,34,35,36], there are still many limitations. Up to date, there is no accessible pathway to obtain accurate arterial wall thicknesses in entire cerebral arteries since the arterial thickness is not uniform as well as patient-specific, which vary along with specific locations and different patients significantly. The simplicity of a uniform arterial thickness and material property, or an assumption of a changing wall thicknesses, has a high probability to introduce new errors/uncertainties to evaluate hemodynamic risks on CAs. 

## 5. Conclusions

In this preliminary study, our findings in a rare pair of identical twins contributed to the knowledge gap in CA pathobiology by studying the configurations and hemodynamics. The morphological and hemodynamic comparisons led to some preliminary conclusions as follows, e.g.,

- Although the twins share identical DNA, identical appearances, and heights, the cerebral arterial morphological configuration and dimensions are different due to non-genetic factors.
- The neck regions of the ruptured aneurysmal sac in twin A-RB present higher TAWSS, and some local luminal surfaces on the sac may have a higher probability to enlarge/rupture given the appearance of a relatively high OSI and low TAWSS. Such a distribution was also observed in the small bleb in twin B-LB.
- Differences in morphologies, although sharing the same DNA information, further lead to varied hemodynamic characteristics in arteries, leading to varying symptoms of CA pathophysiology.
- This preliminary study opens new horizons for the future statistical evaluation of the role of homodynamic parameters on CA pathophysiology.

## Figures and Tables

**Table 1:** Mesh information of the reconstructed MT models for mesh independence tests.

Models	Mesh Name	Min. Size (mm)	Max. Size (mm)	Volume Elements	Volumetric Max. Skewness	Prism Layers	Peel Layers	Size Growth Rate
Twin A-RB	Mesh 01	0.1	1.2	670,515	0.74	10	3	1.05
	Mesh 02 (Final)	0.05	0.8	2,372,078	0.76	10	3	
	Mesh 03	0.03	0.5	6,308,487	0.72	10	3	
Twin A-LB	Mesh 04	0.1	1.2	634,282	0.37	10	3	
	Mesh 05 (Final)	0.05	0.8	1,783,417	0.76	10	3	
	Mesh 06	0.03	0.6	4,000,928	0.65	10	3	
Twin B-LB	Mesh 07	0.1	1.2	716,592	0.81	5	1	
	Mesh 08 (Final)	0.05	0.8	2,606,817	0.8	10	3	
	Mesh 09	0.03	0.6	7,526,671	0.79	10	3	

**Table 2:** Comparisons in ICA dimensions in the LB of MTs based on a perspective view on the plane YZ.

Locations		*a*	*b*	*c*	*d*	*e*	*f*	*g*	*h*
Dimensions (mm)	Twin A	5.28	5.37	4.65	4.09	4.30	4.33	4.31	3.62
	Twin B	5.88	5.49	5.11	4.92	5.15	5.45	3.96	4.33
*Relative Difference* (%)		11.36	2.23	9.89	20.29	19.77	25.86	−8.12	19.62

## Data Availability

Data available on request due to restrictions, e.g., privacy or ethics.
